# Medical History from the Earliest Times

**Published:** 1892-04-16

**Authors:** E. T. Withington


### Medical History from the Earliest Times.

By E. T. Withington, M.A., M.B. Oxon.

### IV.—MEDICINE IN ANCIENT EGYPT.

Among the "mastabas" or tombs of Egyptian grandees which surround the pyramids of Sakkarah is one, small and unpretending in appearance, but remarkable for the beauty and perfection of its inscription. This shows the tomb to be that of Sechmetenanch, chief physician to the Pharaoh Sahura of the fifth dynasty, B.C. 3533 ? and describes how he had healed the king's "nostrils," for which his Majesty wishes him "a long life in holiness." "Then the chief physician spoke before Pharaoh, 'May it please thy soul beloved of Ka, that there be given me a limestone slab like a door for this my tomb in the West-land.' Then the king commanded, and they brought unto him two stone slabs like a double door from the quarry Ko'an, and they were set up in the court of his palace Chaurert-Sahura. The chief taskmaster made the temple masons inscribe them as for the king himself. The Court visited them daily. His Majesty ordered the inscription to be done over with blue-stone." This appears to be the first mention of a physician in history; for, though the older Egyptian dates are uncertain—that given above being the one accepted by Brugsch and the British Museum authorities—few modern Egyptologists would place the fifth dynasty later than B.C. 3000, and we may safely assert that the interval between Sekhet'enanch and Hippocrates was at least as great as that which separates the "Father of Medicine" from our own day. Physicians, as a class, are referred to in still more ancient inscriptions under the term "snu," a word represented hieroglyphically by what seem to be a lancet and cupping instrument.

Who were these first practitioners of what may be called civilised medicine ? The generally accepted theory is that all early physicians, and especially the Egyptian, were priests; but, though in later times the practice of medicine in Egypt was undoubtedly confined to the sacerdotal class, this was by no means necessarily the case at the period now under consideration. Society had then a more patriarchal character—everyone was in some sense a priest—the judges of the goddess of Truth. The officials of the god, specially worshipped by the reigning Pharaoh, and the physicians of Sechmet, the lion-headed goddess of war, to whom, in these earliest ages, the origin of medicine was attributed; but their civil offices were always the most prominent, and there was in addition a separate sacerdotal class to whom the highest and lowest religious functions were entrusted. The disappearance of the "lay element" and the development of this sacerdotal class till it absorbed the professions of scribe, judge, and physician, and finally invaded the throne, itself form the keynote of Egyptian history. The Egyptian physician as met with in Greek writers is primarily a priest, but his special divinity is no longer the war goddess, but Thoth, the god of wisdom and writing ; he has thrown away his lancet and cupping-horn, and abhors bleeding, for do not the sacred writings declare that the blood is the life ? and these writings he must follow in all things, for, should a patient die after treatment not in harmony with them, his own life may pay the penalty.

Returning to the freer times of the old empire, we must travel far down the ages after Sahura before meeting with another interesting relic of Egyptian medicine. This is the domestic medicine chest of the wife of the Pharaoh Mentu'hotep of the eleventh dynasty, about B.C. 2500, not many centuries before Abraham; it contain six vases, one of alabaster and five of serpentine, with dried remnants of drugs, two spoons, a piece of linen cloth, and some roots, enclosed in a basket of straw-work, the whole standing in a wooden chest found in the queen's tomb. We may a'so mention here, as indicating the less formal character of the older medical practice, a curious letter from a husband to his dead wife, found in the latter's tomb. In it he upbraids the departed spirit for having produced disease in him, and while reproachfully calling to mind his kindnesses to her during life, thus describes her last illness. "When thou wast sick, with the sickness that thou hadst, did not I go to the physician and bid him make thy medicines for thee—yea, he did all things whatsoever thou wouldst have him do." This seems to imply that the lady to some extent directed her own treatment, and reminds us of the Homeric description of Egypt as the land where "each one is a physician, skilful beyond all men, for verily they are of the race of Paeon." But our chief source of information is the "Ebers' Papyrus," which, according to its discoverer, is not only the first-known medical document, but the most ancient *complete* book in existence. It was written about 1550 B.C.—some time before the Exodus, though much of its contents is of far greater antiquity; and we have not only the entire work, but also the marginal notes of its owner, who has marked with a large "Good" those prescriptions of which he approves, while the original compiler has added notes of his own, such as "This is a genuine remedy," or "Excellent, I have often seen it, and also made it." But, though mainly a collection of receipts, the "Papyrus" also contains passages on anatomy and diagnosis, the chief of which is the chapter on the heart by a certain Nebsecht, whom Professor Ebers introduces into his interesting romance, "Uarda." The vessels are said to run in pairs, in a manner very similar to that described by the early Greek physicians, though not much in accordance with nature, and to contain not only blood, but air, water, milk, and other fluids. In the doctrine that the heart is the centre of the vascular system, and in the importance attributed to the pulse, the old Egyptian is in advance of Hippocrates himself. "If the physician places his finger on the head, neck, hands, arms, feet, or body, everywhere he will find the heart (*i.e.*, the pulse), for its vessels go to all parts." As examples of diagnosis, we may take the description of four forms of obstruction or constipation. "When thou findest a man with an obstruction—with pale face and beating heart—and findest on examination that his heart is hot and his belly swollen, that is an inflammation (?) that cometh from irritant food. Treat it with something that cools heat and opens the bowels, especially with a draught of sweet beer, poured upon dry neqant fruit. Four times shall he eat or drink (it)." Other forms are that in which "the stomach moves to and fro under the fingers like oil in a bag," that where the patient "vomits and feels very ill," and that in which the belly is "hot and swollen," for each of which different treatment is required. Of prognosis, so prominent in the Hippocratic writings, there is little mention, but here is an example : "If a new-born baby cries 'mj' he will live, if 'mbe' he will die."

We cannot here discuss the multitude of prescriptions contained in this most ancient of medical books; two examples may suffice : To soothe a crying baby a mixture of fly-dung with seeds of the plant Schepen is recommended, which may have been only too effectual if, as is probable, schepen is the poppy. Nor is it impossible that this may be the composition of that soothing drug "Nepenthe," which Polydamna, "wife of Thone, in Egypt, gave to Jove-born Helena," and which has so much puzzled Homeric scholars.

To conclude with a prescription, probably used more frequently than any other: "Against all kinds of witchcraft—a large beetle; cut off his head and wings, boil him, put him in oil, and apply to the part. Then cook his head and wings, put them in serpent's fat, warm it, let the patient drink it."

